# Fees in New York City

**Published:** 1875-09

**Authors:** 


FEES IN NEW YORK CITY.—A New York letter to the Springfield *Republican* gives the following as an account of the incomes of medical men:

"A physician in good practice will receive patients at his office four hours daily, and make calls for about the same length of time. From ten to twenty callers, and half as many house patients, would be a fair average; the fees would be two and five dollars each. At these figures it would not be hard to make up an income of $20,000 or more. It is said of Dr. Willard Parker, I believe, that having been called out of town to attend a patient, he returned a bill of $300, and when it was disputed he showed by his books that his daily receipts were much over that sum. Surgeons' single charges are larger than those of physicians, though the incomes of the latter are probably the highest. For ordinary attendance their rates are about the same, or say five dollars a visit. From twenty-five dollars upwards is the charge for operations. For setting an arm or leg $250 would be asked; larger undertakings being in proportion. For a case requiring a delicate operation and six weeks constant attendance, sometimes two or three times a day, $1,000 was lately asked by a leading surgeon. In another instance, where a wealthy gentleman was badly jammed by a railroad car, he was attended by Dr. James R. Wood, who made about a dozen visits, without any important operation, and sent in a bill of $2,500, which was paid. This is exceeded by Dr. Carnochan, who charged $2,000 for an operation alone, while another surgeon is said to have received $4,500 from one patient.

